# Integrated Assays of Genome-Wide Association Study, Multi-Omics Co-Localization, and Machine Learning Associated Calcium Signaling Genes with Oilseed Rape Resistance to *Sclerotinia sclerotiorum*

**DOI:** 10.3390/ijms25136932

**Published:** 2024-06-25

**Authors:** Xin-Yao Wang, Chun-Xiu Ren, Qing-Wen Fan, You-Ping Xu, Lu-Wen Wang, Zhou-Lu Mao, Xin-Zhong Cai

**Affiliations:** 1 Key Laboratory of Biology and Ecological Control of Crop Pathogens and Insects of Zhejiang Province, Institute of Biotechnology, College of Agriculture and Biotechnology, Zhejiang University, Hangzhou 310058, China; lucymorningstar@foxmail.com (X.-Y.W.); rcx8398@163.com (C.-X.R.); 12216090@zju.edu.cn (Q.-W.F.); 3210103321@zju.edu.cn (L.-W.W.); 3210102846@zju.edu.cn (Z.-L.M.); 2 Centre of Analysis and Measurement, Zhejiang University, 866 Yu Hang Tang Road, Hangzhou 310058, China; ypxu@zju.edu.cn; 3 Hainan Institute, Zhejiang University, Sanya 572025, China

**Keywords:** GWAS, machine learning, quantitative disease resistance, *Sclerotinia sclerotiorum*, calcium signaling

## Abstract

*Sclerotinia sclerotiorum* (Ss) is one of the most devastating fungal pathogens, causing huge yield loss in multiple economically important crops including oilseed rape. Plant resistance to Ss pertains to quantitative disease resistance (QDR) controlled by multiple minor genes. Genome-wide identification of genes involved in QDR to Ss is yet to be conducted. In this study, we integrated several assays including genome-wide association study (GWAS), multi-omics co-localization, and machine learning prediction to identify, on a genome-wide scale, genes involved in the oilseed rape QDR to Ss. Employing GWAS and multi-omics co-localization, we identified seven resistance-associated loci (RALs) associated with oilseed rape resistance to Ss. Furthermore, we developed a machine learning algorithm and named it Integrative Multi-Omics Analysis and Machine Learning for Target Gene Prediction (iMAP), which integrates multi-omics data to rapidly predict disease resistance-related genes within a broad chromosomal region. Through iMAP based on the identified RALs, we revealed multiple calcium signaling genes related to the QDR to Ss. Population-level analysis of selective sweeps and haplotypes of variants confirmed the positive selection of the predicted calcium signaling genes during evolution. Overall, this study has developed an algorithm that integrates multi-omics data and machine learning methods, providing a powerful tool for predicting target genes associated with specific traits. Furthermore, it makes a basis for further understanding the role and mechanisms of calcium signaling genes in the QDR to Ss.

## 1. Introduction

*Sclerotinia sclerotiorum* (Ss) is a notorious plant pathogen capable of infecting over 700 species of monocotyledonous and dicotyledonous plants. It causes a wide range of crop diseases, including white mold, watery soft rot, and Sclerotinia stem rot (SSR) [1,2]. These diseases pose a substantial and widespread threat to crop production, leading to significant economic losses on a global scale. Among the affected crops is oilseed rape (*Brassica napus*), which holds a prominent position as one of the world’s most vital oilseed crops. For instance, in temperate climates, Ss infections can lead to crop yield reductions of 80–100% [3,4]. Ss presents a major threat due to its ability to infect diverse plant species and the absence of effective host resistance mechanisms, thereby lacking stable resistant cultivars. Consequently, understanding the molecular mechanisms of plant resistance to Ss and developing resilient cultivars have become critical objectives in agricultural research. Quantitative disease resistance (QDR) has emerged as a key component in the defense against Ss in oilseed rape and other crops. QDR involves the cumulative effects of multiple quantitative trait loci (QTLs) that collectively contribute to resistance [5,6]. The complexity and diversity of QDR mechanisms necessitate further investigation into its genetic foundations and evolutionary aspects. Unraveling the molecular architecture of QDR reveals an intricate network that integrates multiple response pathways, incorporating various pathogen molecular determinants and environmental cues [7,8]. Multiple studies in the past decade have identified Ss resistance-related QTLs across various chromosomes. These include QTL SRC6 on chromosome C06 containing the candidate gene *BnaC.IGMT5.a* belonging to the monolignol biosynthetic gene family [9]; QTL DSRC4 on chromosome C04 with two tau class glutathione S-transferase (GSTU) genes *GSTU3* and *GSTU4* [10]; and another QTL carrying a GSTU gene cluster on chromosome C06 [11].

In addition to QTL, genome-wide associated study (GWAS) was employed to identify genes involved in QDR to Ss. The QTLs predicted by parental linkage analysis are typically restricted to the differences between specific parental lines, with limited capacity to explore diversity in large-scale and more diverse populations [12]. In contrast, GWAS based on population structure can capture a broader range of genetic variations, aiding in the better understanding of the polygenic genetic background underlying complex quantitative traits. GWAS, employing high-density single nucleotide polymorphisms (SNPs) markers, allows for more precise gene localization, thereby facilitating the identification of individual loci associated with quantitative resistance [13]. Using GWAS, *BnaA08g25340D* (*BnMLO2_2*) and *BnaC07g35650D* (*BnGLIP1*) were identified to be associated with SSR resistance in *B. napus*, which was validated by Arabidopsis mutant inoculation assays [6,14]. In summary, only a very limited number of genes associated with QDR to Ss have been identified using QTL and GWAS assays. Further efforts are required to identify more important loci and genes associated with QDR to Ss.

GWAS and post-GWAS approaches have been developing to predict genome-wide loci and genes associated with an interesting trait. For GWAS, in addition to single-SNP-based GWAS (single-SNP GWAS), haplotype-based GWAS (HAP-GWAS) was developed for better capturing long-range linkages [15,16]. Moreover, post-GWAS technologies such as omics-wide association studies (OWAS) have been continuously developed to obtain more accurate and reliable genes of interest [17]. These include epigenome-wide association studies (EWAS) for epigenomics [18], transcriptome-wide association studies (TWAS) for transcriptomics [19], and metabolome-wide association studies (mGWAS) for metabolomics data [20]. Weighted gene co-expression network analysis (WGCNA) and expression quantitative trait nucleotide (eQTN) co-localization can also provide more accurate predictions [21]. Composite resequencing-based GWAS combines conventional GWAS with rare allele testing, functional prediction, and prior knowledge [22]. Integrated assays of various GWAS and post-GWAS approaches should result in more accurate and reliable predictions especially for complex traits such as QDR.

Machine learning (ML) methods have emerged as powerful tools for handling and analyzing high-dimensional datasets to capture nonlinear relationships within genotypes [23,24]. In recent years, an increasing number of studies have utilized ML in the identification of phenotypes-associated target genes [25,26,27]. The algorithms QTG-Finder and QTG-Finder2 have been developed based on Random Forest, trained using known causal genes from different species, and utilize features such as polymorphism, functional annotation, and co-functional networks to prioritize genes within QTLs [28,29]. QTG-Finder does not test GWAS results but predicts genes within known QTLs. The important features for identifying causal genes through QTL mapping may differ from those identified through GWAS. QTL mapping tends to identify large-effect alleles in protein-coding regions, while GWAS tends to identify common alleles with larger effect sizes in both protein-coding and non-coding regions [30]. In oilseed rape, the POCKET algorithm [31] has been developed to predict target genes associated with seed oil content by integrating multi-omics features, including TWAS results. To date, no algorithm is available that efficiently integrates multiple post-GWAS results to rapidly predict disease-resistant genes within resistance-associated loci (RALs), which are specific regions on the chromosome containing a large number of SNPs associated with disease resistance. Current research relies partially on specific features that may not fully explain the variations identified by GWAS, thereby limiting their applicability across different traits. Novel algorithms capable of effectively integrating a broader range of omics information to rapidly predict target genes associated with QDR within larger regions remain to be developed.

Cellular calcium ion concentration ([Ca^2+^]) serves as a ubiquitous second messenger, widely present from prokaryotes to eukaryotes, and plays a crucial role in plant growth and development, and biotic and abiotic stress responses [32,33,34]. The regulation of calcium influx is accomplished by calcium channels and pumps, such as glutamate receptor (GLR), cyclic nucleotide-gated channels (CNGC), and Ca^2+^/H exchangers (CAX) [33,35]. Calcium sensors, including calmodulin (CaM), calmodulin-like proteins (CML), calcium-dependent protein kinases (CDPK), and calcineurin B-like proteins (CBL), perceive changes in intracellular calcium concentration and activate downstream kinases. These kinases phosphorylate regulatory proteins, such as transcription factors or transporters/channels, thereby directly modulating gene expression or transporter/channel activity, leading to stress tolerance, plant adaptation, and other phenotype responses [36,37]. Which calcium signaling genes are involved in the QDR to Ss remains unclear.

This study aims to identify on a genome-wide scale the genes associated with the QDR to Ss in oilseed rape, providing a molecular basis for breeding resistant varieties. We identified 48 RALs associated with resistance to Ss through single-SNP GWAS, and co-localized seven highly correlated RALs associated with this resistance employing HAP-GWAS in conjunction with WGCNA and RNA-Seq. Furthermore, we developed Integrated Multi-Omics Analysis and Machine Learning for Target Gene Prediction (iMAP), a machine learning algorithm based on Random Forest (RF), incorporating multi-omics features, to predict optimal target genes associated with Ss resistance. Consequently, we successfully identified a set of calcium signaling genes exhibiting evolutionary selection and breeding potential for resistance to Ss.

## 2. Results

### 2.1. Optimization for Improved Single-SNP GWAS for SSR Resistance in Oilseed Rape

**Figure 1:**
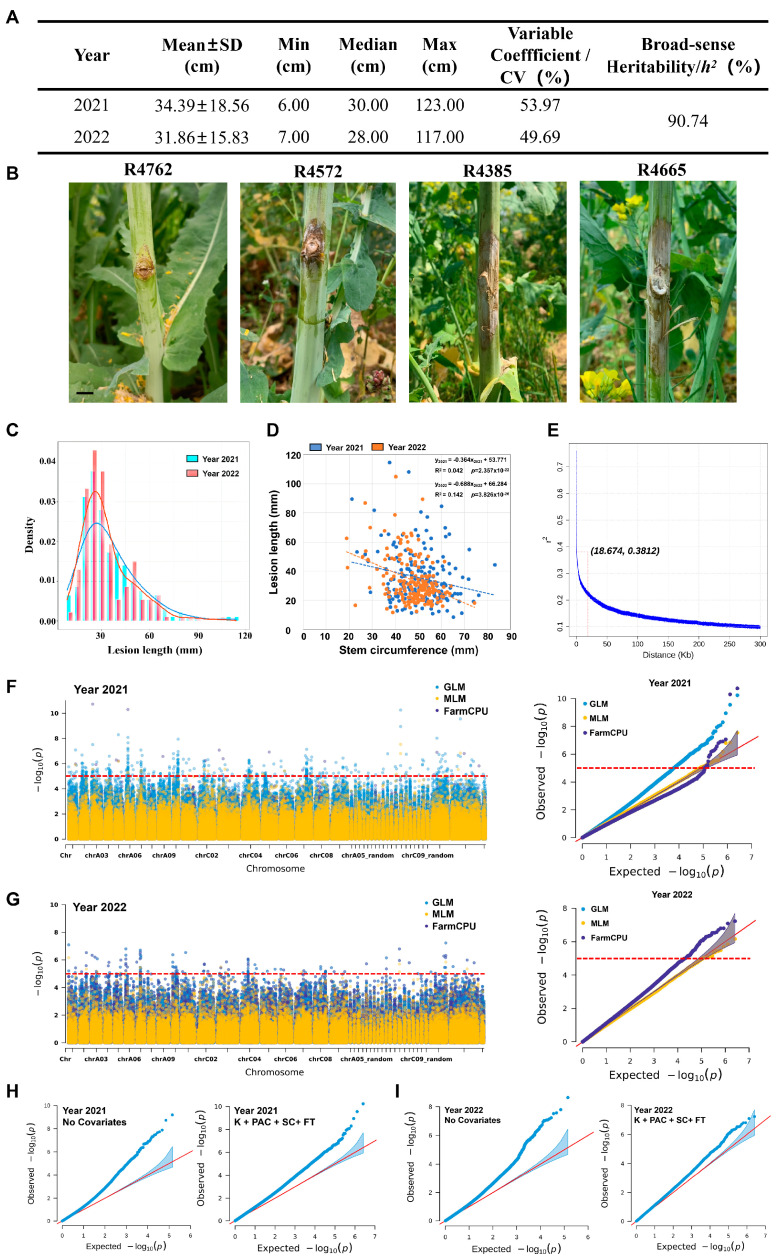
Optimized GLM-based single-SNP GWAS analysis with covariates for SSR resistance in oilseed rape. (**A**) Phenotypic variation of 300 oilseed rape germplasm accessions in resistance to sclerotinia stem rot (SSR). Lesion length was measured 14 days post inoculation (dpi) in the years 2011 and 2022. (**B**) The disease symptoms of oilseed rape stems after Ss inoculation. Representative resistant (R4762 and R4572) and susceptible (R4385 and R4665) rapeseed germplasm accessions are shown. Bar = 1 cm. (**C**) Frequency distribution of lesion length in genome-wide association study (GWAS) population in years 2021 and 2022. (**D**) Regression analysis between stem circumference (SC) and stem lesion length (LL) in respect to SSR resistance. (**E**) Linkage disequilibrium (LD)-decay plot. LD (r^2^) was estimated with PopLDdecay v3.42 and plotted as a function of physical distance in Kb for each population. (**F**,**G**) Multi-track Manhattan plot and Quantile–Quantile (Q-Q) plot based on single-SNP GWAS using GLM, MLM, and FarmCPU models for the phenotypic data collected in 2021 (**F**) and 2022 (**G**). The red dashed lines indicate the significance threshold (−log_10_ (*p*_value) = 5.0). (**H**,**I**) Q-Q plots comparing single-SNP GWAS results with and without covariates were generated in years 2021 (**H**) and 2022 (**I**). The covariates kinship (K), Principal Component Analysis (PCA), SC, and flowering time (FT) were included.

To evaluate the filed resistance of oilseed rape resources to Ss, we conducted two-year field inoculation analyses for 300 oilseed rape accessions in Changxing, China in 2021 and 2022. The length of stem lesions (LL) and the corresponding stem circumference (SC) were measured two weeks after stem inoculation with Ss mycelial plugs. Although there were slight differences in the maximum, minimum, and median values of lesion lengths between the two years, the overall trend was consistent. The broad-sense heritability (*h*^2^) of lesion length was 90.74%, indicating that the stem resistance of the accessions to Ss is genetically stable and independent of the environment (Figure 1A). Consequently, two-year consistently resistant (e.g., R4762 and R4572) and susceptible (e.g., R4385 and R4665) rapeseed germplasm collections were identified (Figure 1B). Furthermore, the lesion length data for both years followed a normal distribution. Correlation analysis between stem circumference and lesion length revealed a weak negative relationship (Figure 1C,D). GWAS was performed based on the reported single-SNP data for the collected 300 accessions [38], in which the distribution of SNPs across all chromosomes of oilseed rape was illustrated in Figure S1. Linkage disequilibrium (LD) decay analysis was performed, and a distance of 18.674 kb, where r^2^ decreased by half, was selected (Figure 1E). Three models MLM, GLM, and FarmCPU for GWAS using the R package rMVP v1.0.0 [39] were compared. The results for the two-year data showed that the GLM model demonstrated better control over false positives and false negatives, making it the most suitable model for this experiment (Figure 1F,G). Fifty principal components (PCs) were calculated using Plink v1.9, and significant tests were performed using EIGENSOFT v6.0.1. After conducting the significance tests, the first 16 highly significant PCs were selected as covariates (Table S1). The inclusion of kinship (K), Principal Components Analysis (PCA), SC, and flowering time (FT) significantly reduced false positives (Figure 1H,I). Finally, the GLM model, incorporating K, PCA, SC, and FT, was considered the optimal approach for conducting single-SNP-based GWAS (single-SNP GWAS) for candidate resistance gene identification.

### 2.2. Identification of Resistance-Associated Loci and Candidate Genes by Optimized Single-SNP GWAS

**Figure 2:**
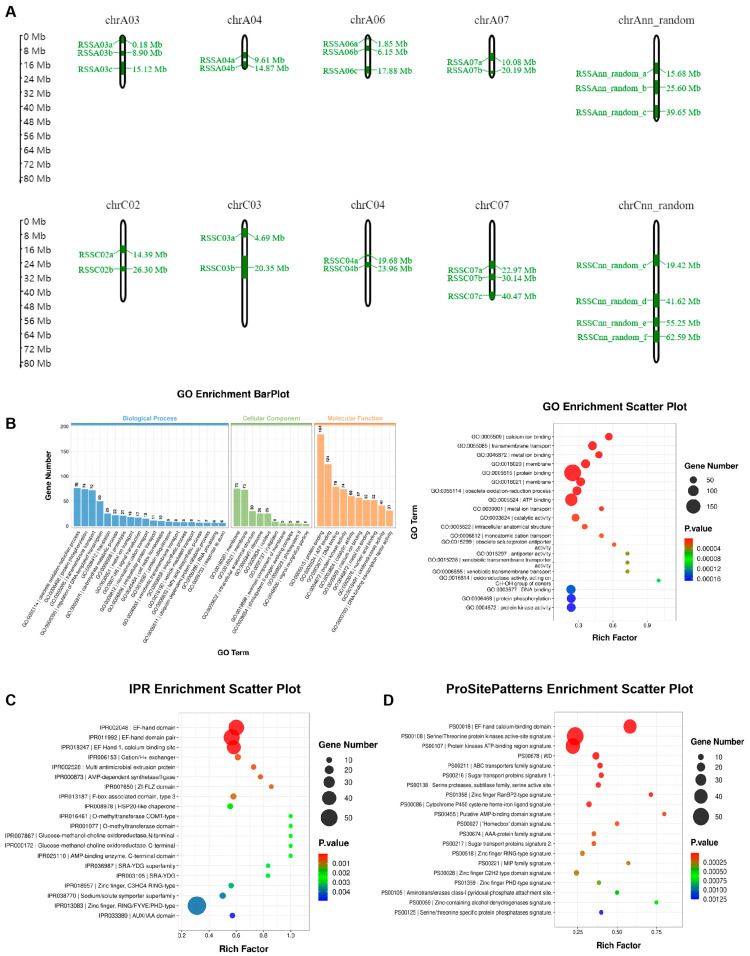
Genetic analysis and functional characterization of 48 SSR resistance-associated RALs in Oilseed Rape. (**A**) Chromosomal distribution of 24 out of the total 48 SSR resistance related resistance-associated loci (RALs). The distribution of the remaining RALs can be found in Figure S2. (**B**) The results of gene ontology (GO) enrichment analysis of the genes within the 48 SSR resistance-associated RALs. (**C**,**D**) Protein structure analysis of genes within the 48 SSR resistance-associated RALs based on IPR (**C**) and ProSitePatterns (**D**) databases.

The single-SNP GWAS for the SSR in oilseed rape was performed using our optimized parameters described above. We filtered significant SNPs from the single-SNP GWAS results (−log_10_(*p*_value) > 5) and consequently identified 48 resistance-associated loci (RALs) associated with resistance to Ss (with at least three significant SNPs per 18 MB) by referring to the range of previously identified Ss resistance-associated quantitative trait loci (QTLs) in existing studies. Among these 48 RALs, 15 overlapped with previously reported QTLs associated with Ss resistance, while the remaining 33 were newly discovered (Table 1). The Ss-associated RALs were found to be distributed across multiple chromosomes, including A03, A06, C05, C07, Ann_random, and Cnn_random. Each of these chromosomes contained at least three or more RALs associated with resistance to Ss (Figure 2A and Figure S2). The gene ontology (GO) database was used to annotate and enrich the functional characteristics of the genes within the 48 Ss-associated RALs (Figure 2B; Table S2). The analysis revealed that many genes were enriched in various biological processes, including obsolete oxidation-reduction process, protein phosphorylation, transmembrane transport, and the regulation of DNA-templated transcription. In terms of cellular components, the majority of genes were located in the membrane, while others were distributed in intracellular anatomical structures, ribosomes, and the nucleus. Regarding molecular functions, several genes were significantly enriched in various functional processes, such as protein binding, ATP binding, DNA binding, protein kinase activity, catalytic activity, calcium ion binding, metal ion binding, and nucleic acid binding. Further analysis of gene structure and enrichment using the IPR database (Figure 2C and Figure S3A; Table S3) and ProSitePatterns database (Figure 2D and Figure S3B; Table S4) revealed that many genes contained structural domains associated with calcium ion binding, such as the EF-hand domain, EF-hand domain pair, and EF-Hand 1, calcium-binding sites. Additionally, some genes contained structural domains related to protein kinase activity, such as Serine/Threonine protein kinases active-site signature and Protein kinases ATP-binding region signature. In summary, the gene functional annotation and enrichment analysis of the 48 SSR resistance-associated RALs revealed the potentially important roles of these genes in the QDR to Ss.

### 2.3. Identification of RALs and Genes by Integrated Assays of Single-SNP GWAS, Hap-GWAS, WGCNA, and DEGs

**Figure 3:**
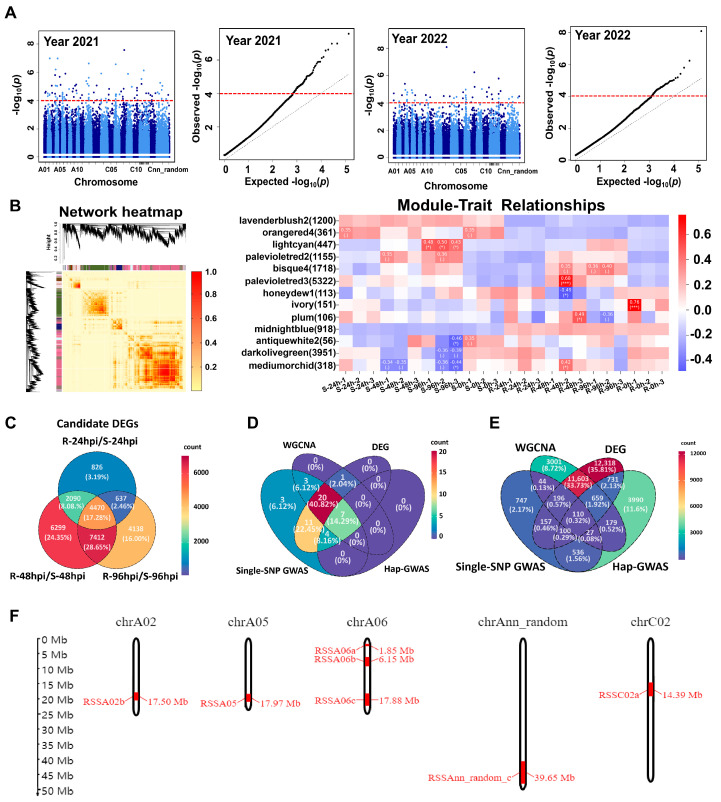
Multi-Omics analysis reveals the co-predicted SSR resistance-associated RALs in Oilseed Rape. (**A**) Manhattan and Q-Q plots for haplotype-based GWAS (HAP-GWAS) for Ss resistance in oilseed rape for the years 2021 and 2022. The red dashed lines indicate the significance threshold (−log_10_ (*p*_value) = 4.0). (**B**) Weighted gene co-expression network analysis (WGCNA) for gene expression in responsive to Ss infection in susceptible and resistant oilseed rape germplasm accessions. Correlation heatmap of disease resistance modules was shown. Module colors in coordinates of the left panel correspond to those in Y-axis of the right panel. The Pearson correlation algorithm in OECloud tools (https://cloud.oebiotech.com, accessed on 6 September 2023) was used to calculate the correlation coefficient and *p* value of the module’s characteristic genes and traits. “.” indicates non-significance. * *p* ≤ 0.05; *** *p* ≤ 0.001. (**C**) Venn diagrams illustrating the overlap of differentially expressed genes at 24 hpi, 48 hpi, and 96 hpi after *Sclerotinia* infection in susceptible and resistant oilseed rapes. (**D**) Venn diagrams depicting the overlapping of SSR resistance-associated RALs identified through single-SNP GWAS, HAP-GWAS, WGCNA, and differentially expressed genes (DEGs) analyses. (**E**) Venn diagrams illustrating the overlapping of SSR resistance-associated genes identified through single-SNP GWAS, HAP-GWAS, WGCNA, and DEGs analyses. (**F**) Chromosomal distribution of 7 SSR resistance-associated RALs that are unanimously identified in single-SNP GWAS, HAP-GWAS, WGCNA, and DEG analyses.

Haplotype-based GWAS (HAP-GWAS) has been considered a better predictor of reliable RALs [15,16]. Therefore, Hap-GWAS was further used to analyze the two-year stem inoculation results in oilseed rape (Figure 3A). It identified 11 RALs that overlapped with the results from the single-SNP GWAS. Moreover, weighted gene co-expression network analysis (WGCNA) was performed on RNA-Seq data obtained from susceptible and resistant rapeseed germplasm accessions following stem inoculation with Ss (NCBI Sequence Read Archive, accession no. SRP053361) (Figure S4). The analysis revealed 13 significantly upregulated modules (Figure 3B), and within these modules, 30 RALs were co-located with the results from single-SNP GWAS. Similarly, the same batch of RNA-Seq data [10] was used to analyze differentially expressed genes (DEGs) at three time points: 24 hpi, 48 hpi, and 96 hpi. A total of 4470 genes were co-located (Figure 3C), indicating their potential significance as candidate genes associated with resistance to Ss. By intersecting the significant disease-resistant genes from single-SNP GWAS, Hap-GWAS, WGCNA, and DEGs, a total of 7 RALs and 110 potential target genes were identified (Figure 3D,E). The specific locations of these RALs on the chromosomes are illustrated in Figure 3F. Interestingly, three RALs were found on chromosome A06, appearing in both GWAS, WGCNA, and DEGs analyses. This suggests that chromosome A06 may play a crucial regulatory role in resistance to Ss in oilseed rape. Consequently, A06 became the focal point for further in-depth investigation.

### 2.4. iMAP Predicts the Involvement of Calcium Signaling Genes in Resistance to Ss

**Figure 4:**
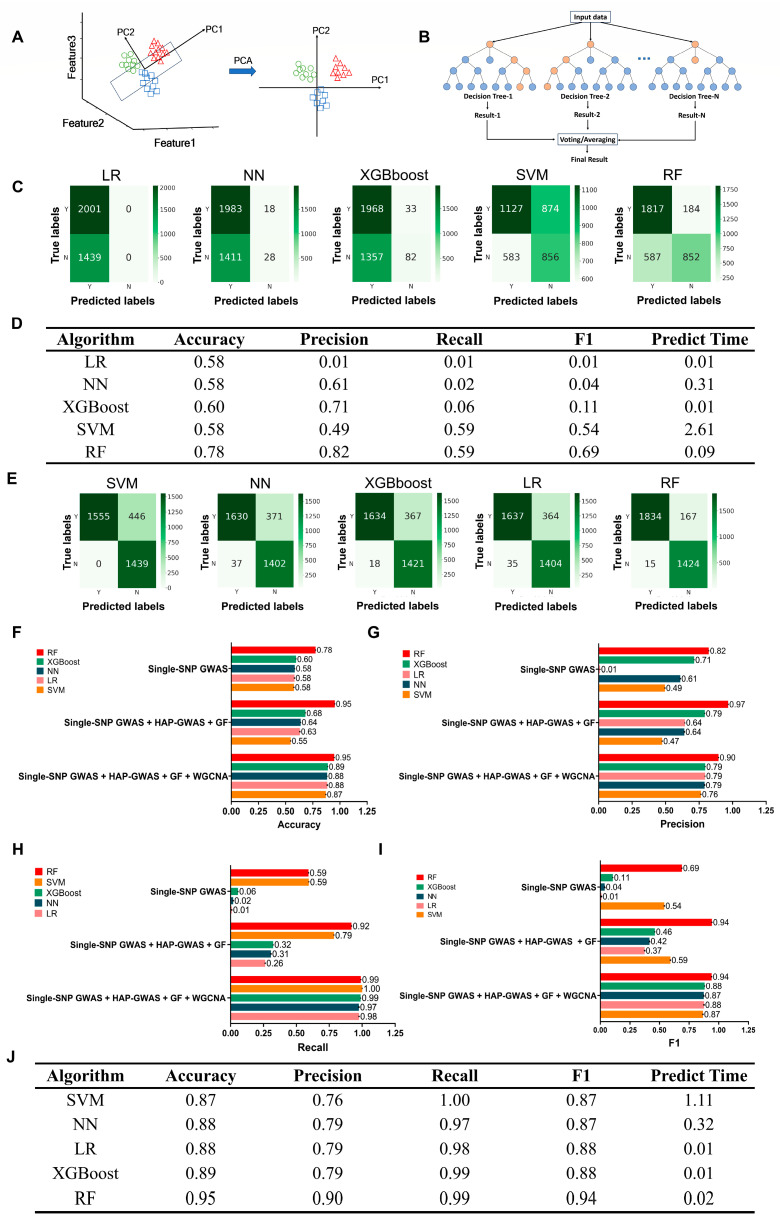
Exploration of algorithms and feature sets for genomic analysis in predictive modeling. (**A**) Illustration of PCA algorithm-mediated reduction of data dimensions from a three-dimensional plane (left) to a two-dimensional plane (right). Different groups of data are indicated in various colors. (**B**) Work model of the Random Forest (RF) algorithm, combining multiple decision trees with randomly selected data sets and features. (**C**) Confusion Matrix of Logistic Regression (LR), Support Vector Machine (SVM), eXtreme Gradient Boosting (XGBoost), Neural Network (NN) and RF algorithms using single-SNP GWAS as the feature set. (**D**) Performance comparison of LR, NN, XGBoost, SVM, and RF algorithms using single-SNP GWAS as the feature set in terms of accuracy, precision, recall, F1 score, and predict time. (**E**) Confusion Matrix of SVM, NN, XGBoost, LR, and RF algorithms using single-SNP GWAS+ HAP-GWAS + gene function (GF) + WGCNA as the feature set. (**F**–**I**) Performance evaluation of LR, NN, XGBoost, SVM, and RF algorithms in terms of accuracy (**F**), precision (**G**), recall (**H**), and F1 score (**I**) using different feature sets, including single-SNP GWAS, single-SNP GWAS + HAP-GWAS + GF, and single-SNP GWAS + HAP-GWAS + GF + WGCNA for machine learning. (**J**) Performance comparison of LR, NN, XGBoost, SVM, and RF algorithms using single-SNP GWAS + HAP-GWAS + GF + WGCNA as the feature set in terms of accuracy, precision, recall, F1 score, and predict time.

To better predict candidate genes related to resistance against Ss within RALs, we collected diverse features and constructed a forward training set to develop a machine learning algorithm, which is named here Integrated Multi-Omics Analysis and Machine Learning for Target Gene Prediction (iMAP). This algorithm combines Principal Component Analysis (PCA) and Random Forest (RF) to achieve accurate predictions. Specifically, we use the dimensionality reduction technique PCA (Figure 4A), which transforms high-dimensional data into a lower-dimensional space while retaining the most important information [44]. RF is a powerful ensemble learning method that combines multiple decision trees (Figure 4B) to improve the accuracy and robustness of predictions [45]. To validate the effectiveness of the RF algorithm, we compared it with Logistic Regression (LR), Support Vector Machine (SVM), eXtreme Gradient Boosting (XGBoost), and Neural Network (NN) algorithms. When using only single-SNP GWAS as features, based on the confusion matrix analysis (Figure 4C,D), RF exhibited the highest accuracy (0.78), while SVM, LR, NN, and XGBoost achieved accuracies of 0.58, 0.58, 0.58, and 0.60, respectively. In terms of precision, RF had the highest value (0.82), indicating that 82% of the predicted positive samples were true positives, while that of the remaining algorithms was lower than 0.71. The recall rate, which measures the model’s ability to correctly identify positive samples, was relatively high for RF and SVM at 0.59. The F1 score, a comprehensive performance metric that combines precision and recall, was highest for RF (0.69), while LR, NN, XGBoost, and SVM had F1 scores 0.01, 0.04, 0.11, and 0.54, respectively. RF, LR, and XGBoost can all perform fast computations with a prediction time below 0.1 s, whereas SVM had the longest prediction time (2.61 s) (Figure 4D). In summary, RF exhibited superior performance in terms of accuracy, precision, recall, and F1 score, along with faster prediction. Therefore, RF appears to be the best-performing algorithm on the given dataset. Furthermore, we conducted tests by expanding the feature set beyond single-SNP GWAS (Figure 4E–J and Figure S5A,B). The addition of HAP-GWAS, gene function (GF), and WGCNA features improved the performance of most classification models. Based on F1 score, precision, recall, and accuracy, the performance of the single-SNP GWAS + HAP-GWAS + GF + WGCNA data combination significantly outperformed that of single-SNP GWAS and single-SNP GWAS + HAP-GWAS + GF combinations. Among all the data combinations, RF consistently performed the best, achieving the highest F1 scores, precision, recall, and accuracy. This indicates that RF possesses strong classification capabilities for handling multiple data combinations.

Finally, using the RF model and the single-SNP GWAS + HAP-GWAS + GF + WGCNA dataset, we predicted seven calcium signaling genes within three key RALs on chromosome A06, namely *CIPK17* (BnaA06g03950D), *SLP2* (BnaA06g12600D), *CPK4* (BnaA06g15970D), *CML15* (BnaA06g12600D), *CML44* (BnaA06g15280D), *IQD30* (BnaA06g13020D), *IQD32* (BnaA06g14070D) (Table 2). These results suggest the important roles of calcium signaling pathways in the QDR to Ss and demonstrate the powerful performance of iMAP in precise prediction of the target genes.

### 2.5. Positive Selection of Multiple Calcium Signaling Genes in the Population Evolution for Ss Resistance in Oilseed Rape

**Figure 5:**
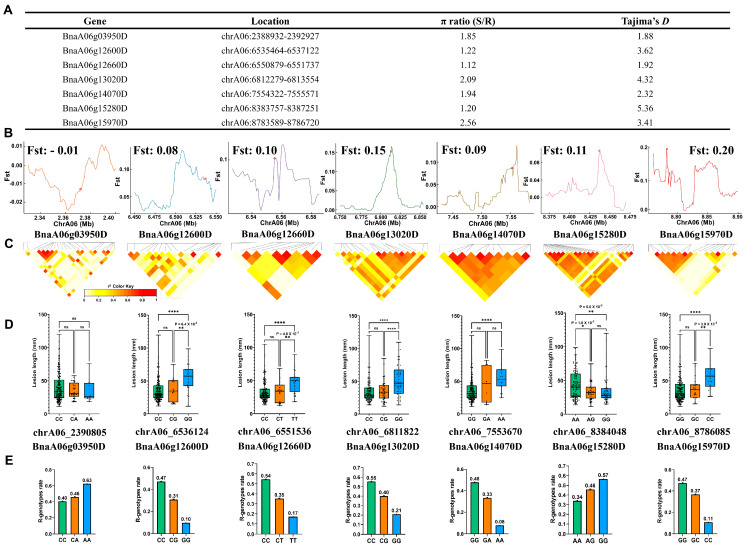
Positive selection on potential key calcium signaling genes associated with Ss resistance in oilseed rape. (**A**) Chromosomal distribution of seven calcium signaling genes associated with SSR resistance, and their genetic diversity (π) levels and Tajima’s *D* between susceptible (S) and resistant (R) subgroups. (**B**) The Fixation Index (Fst) between susceptible (S) and resistant (R) groups for the distribution of seven calcium signaling genes. (**C**) LD plots illustrating the genomic region surrounding the focal SNPs of the six calcium signaling genes. (**D**) The haplotype frequencies for four SNPs in the coding sequence (CDS) and promoter regions of the seven calcium signaling genes. Significant difference was determined by one-way ANOVA followed by DMRT (ns, non-significance; * *p* ≤ 0.05; ** *p* ≤ 0.01; **** *p* ≤ 0.0001). Specific *p*-values are shown in the panels when *p* > 0.01. (**E**) The R-genotypes rate in each of the three genotypes, which is calculated as the proportion of germplasm accessions with a lesion length less than 30 mm out of the total number of germplasm accessions for each genotype.

To further investigate whether the seven calcium signaling genes predicted by iMAP on chromosome A06 have undergone positive selection during the evolution of the oilseed rape population, we conducted nucleotide diversity ratio (π ratio) and Tajima’s *D* statistical analysis on the A06 chromosome in resistance and susceptibility subpopulations (Figure 5A). The π ratio values of the genes ranged from 1.20 to 2.56, indicating a higher level of genetic variation between the resistance and susceptibility subpopulations. Tajima’s *D* values ranged from 1.88 to 5.36, potentially indicating signs of non-neutral evolution, possibly due to positive selection. *BnaA06g15970D* and *BnaA06g13020D* showed higher fixation index (Fst) values of 0.20 and 0.15, respectively, while *BnaA06g12600D*, *BnaA06g12660D*, *BnaA06g14070D*, and *BnaA06g15280D* exhibited moderate Fst values. These findings imply that these genes have experienced positive selection between the resistance and susceptibility subpopulations (Figure 5B). Furthermore, we performed an r^2^ analysis of SNP loci within the gene regions (Figure 5C) and compared lesion lengths among three different genotypes (no munition, single and double nucleotide mutations) of selected SNP to identify the optimal genotypes for disease resistance. Apart from *BnaA06g03950D*, which showed comparable lesion lengths across the three haplotypes, the other six genes (*BnaA06g12600D*, *BnaA06g12660D*, *BnaA06g13020D*, *BnaA06g14070D*, *BnaA06g15280D*, and *BnaA06g15970D*) exhibited significant disparities in lesion length among the genotypes. Additionally, there were significant differences between single and double nucleotide mutations in the SNP loci of these six genes, indicating that these mutations may gradually contribute to changes in disease resistance among germplasm accessions (Figure 5D,E).

In summary, the predicted calcium signaling genes have undergone positive selection during the evolution of the oilseed rape population and may be associated with the evolution of disease resistance in oilseed rape.

## 3. Discussion

SSR caused by the necrotrophic fungal pathogen *Sclerotinia sclerotiorum* is an economically important disease in oilseed rape [1,46]. However, resistance to SSR is a complex quantitative disease resistance (QDR), characterized by subtle cumulative and partially dominant effects [11,47]. In contrast to typical resistance mediated by single *R* genes, QDR is controlled by the complex interaction of multiple genes, involving multiple loci and genetic factors, potentially associated with different immune response pathways. QDR exhibits a continuous spectrum of disease resistance phenotypes, indicating that different individuals may display varying levels of resistance, rather than a binary classification of resistant or susceptible [48,49]. GWAS based on linkage disequilibrium (LD) can provide more precise localization of RALs. Several important RALs associated with resistance to Ss have been identified on multiple chromosomes in oilseed rape. These RALs harbor genes involved in oxidative burst, lignin biosynthesis, and jasmonic acid (JA) pathways [11,50,51]. In this study, we identified a total of 48 RALs associated with resistance to Ss, of which 15 RALs were consistent with previous studies. In addition to the well-established A02 and C09 chromosomes, we observed the presence of overlapping RALs on eight other chromosomes, further confirming the repeatability and reliability of the RALs identified in this GWAS. Gene ontology (GO) annotation revealed a significant enrichment of genes associated with calcium ion binding and protein kinase activity, highlighting the potentially important role of the calcium signaling pathway in resistance to Ss.

**Figure 6:**
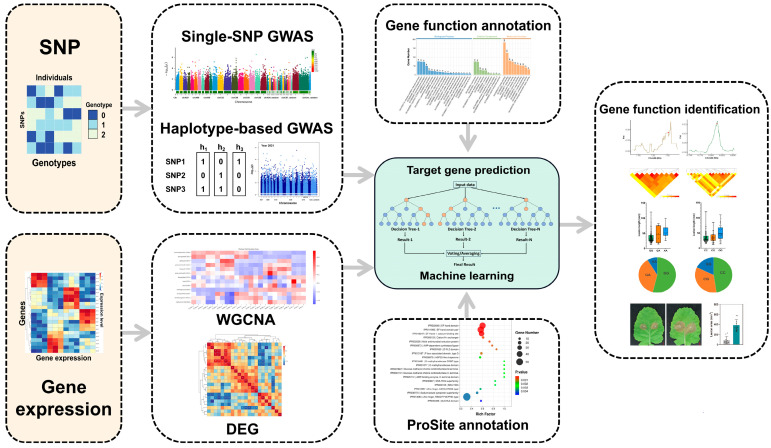
Work model of iMAP. We integrate multi-omics data to perform comprehensive analyses, including Single-SNP GWAS and Hap-GWAS on SNP data, and WGCNA and DEGs analysis on gene expression data. Additionally, iMAP allows for the integration of SNP and expression data for TWAS. Furthermore, we incorporate various databases for functional and structural analysis of genes. By using Random Forest algorithm, iMAP performs machine learning on different features to predict potential target genes associated with traits of interest. These predicted genes can be validated through further biological experiments to explore their functional roles.

Currently, GWAS often involves large genomic regions when predicting QTL. To address this limitation, numerous co-localization strategies that integrate multi-omics data have been developed in this study. Machine learning techniques have the ability to integrate diverse data sources and perform feature selection, enabling the construction of predictive models for target gene prediction and unraveling complex associations between genotypes and phenotypes [52,53]. Random Forest is a powerful ensemble learning algorithm that combines multiple decision trees to create an accurate and robust model. It reduces overfitting, handles high-dimensional data, and improves prediction accuracy through the majority voting of individual decision trees [45,54]. Currently, there have been numerous studies utilizing machine learning and analyzing multi-omics data to identify relevant genes associated with crop yield in economic crops [55]. However, there is still a lack of developed algorithms that can extensively analyze multi-omics data, specifically targeting plant disease resistance, particularly QDR. In this study, we propose a novel approach that integrates multi-omics and machine learning techniques, iMAP, to gain deeper insights into the molecular mechanisms underlying plant disease resistance (Figure 6). The development of the iMAP algorithm has provided researchers with a powerful tool to rapidly rank and list potential candidate genes associated with specific traits within a large number of RAL regions. This lays the foundation for a deeper understanding of gene function and enables advancements in precision breeding and other research areas. Moreover, the algorithm is not limited to specific species or traits and can flexibly incorporate, integrate, and analyze various features based on different research objectives and data characteristics. It demonstrates good performance in terms of F1 score even with limited feature data, highlighting its wide range of potential applications.

In crop improvement, achieving high yields requires finding an appropriate balance between growth and defense, as immune activation often comes with high costs and compromises in growth and development, known as “growth-defense tradeoffs” [56]. Calcium ions (Ca^2+^) play a pivotal role as secondary messengers in various developmental and physiological processes in plants and have long been considered crucial in plant immune responses. While pattern recognition receptors (PRRs) and nucleotide-binding domain leucine-rich repeat proteins (NLRs) are activated by different receptors, their signaling cascades enhance a range of defense responses [31,57]. Recent studies have revealed the molecular functionality of at least some coiled-coil (CC) NLRs (CNLs) and RPW8-like NLRs (RNLs) as calcium-permeable cation channels, further highlighting the importance of calcium in defense mechanisms [58,59]. During the pattern-triggered immunity (PTI) process, BIK1 phosphorylates and activates the CNGC2-CNGC4 channels [60]. Simultaneously, these channels play a crucial role in maintaining intracellular calcium ion balance, preventing the excessive accumulation of cytoplasmic calcium ions, thereby affecting growth and development [61]. In summary, calcium ions play a pivotal role in plant immune responses by regulating multiple signaling pathways and gene expression, thereby influencing plant resistance against pathogens. Components such as calcium channels and calcium-dependent protein kinases are key players in these processes. The activation of plant immunity incurs energy costs and modifies hormone signaling, leading to a defense-growth trade-off [62]. Breeding high-quality economic crops necessitates achieving a delicate equilibrium between yield and pathogen resistance. Recent studies have emphasized the regulatory role of CAXs in intracellular calcium signaling and the attainment of growth-immunity balance [63]. Further investigation is needed to understand the molecular mechanisms underlying the involvement of calcium signaling in plant growth and disease response, which is crucial for improving crop disease resistance while maintaining optimal yield in modern agriculture. Through integrated assays of single-SNP GWAS, Hap-GWAS, WGCNA, and DEGs, we have identified three significant RALs associated with resistance to Ss on chromosome A06 in oilseed rape. Furthermore, using the iMAP algorithm, we predicted seven calcium signaling genes with high relevance to disease resistance: *CIPK17* (BnaA06g03950D), *SLP2* (BnaA06g12600D), *CPK4* (BnaA06g15970D), *CML15* (BnaA06g12600D), *CML44* (BnaA06g15280D), *IQD30* (BnaA06g13020D), and *IQD32* (BnaA06g14070D). Some members of these gene families have already been identified in other crops for their crucial roles in resistance against different pathogens and the regulation of plant growth. CML8 in Arabidopsis positively regulates immune responses against *Pseudomonas syringae* associated with the salicylic acid (SA) signaling pathway [64]. In wheat, the overexpression of CIPK14 enhances broad-spectrum resistance against wheat stripe rust [65], and TaCIPK15-4A plays a positive role in wheat resistance against powdery mildew [66]. CBL-CIPK complexes play a role in seed germination and protect seeds and germinating seedlings from salt stress through the CBL5-CIPK8/CIPK24-SOS1 pathway [67]. Furthermore, we have previously found that some components of calcium signaling pathways are involved in plant resistance to Ss. These include calcium generators guanylate cyclase (GC) [68] and CNGCs [69,70], Ca^2+^ sensors CaM2 and CaM6 [71], and CDPK, as well as CRK and Ca^2+^/CaM-dependent protein kinase (CCaMK) [72,73], and calcium signaling relays the transcription factor CAMTA3 [74,75]. These results not only verify the prediction results in this study and thus demonstrate the power of the approaches developed in this study to identify the target genes, but also highlight the potentially pivotal roles of calcium signaling pathways in the QDR to Ss.

Nevertheless, further experiments in oilseed rape are required to confirm the functions and elucidate the mechanisms of these calcium signaling genes in resistance to Ss. These calcium signaling genes, which may regulate calcium ion concentrations and signaling pathways, modulate plant growth rhythms, nutrient allocation, and energy utilization to achieve an effective balance between growth and defense in response to diverse growth environments and biotic pressures. An in-depth investigation of the molecular mechanisms and regulatory networks involved in calcium signaling homeostasis can enhance crop adaptability, disease resistance, and yield stability, contributing to sustainable agriculture and food security.

## 4. Materials and Methods

### 4.1. Plant Cultivation and Field Inoculation

The oilseed rape (*Brassica napus*) germplasm accessions used in this study were sourced from the core germplasm as described [38]. A total of 300 oilseed rape accessions from 39 countries were cultivated in Changxing, China, during the years 2021 and 2022. The experiment consisted of three replicates, with each replicate containing more than 16 plants of each variety. Within each replicate, three randomly selected plants of each variety were inoculated with stem inoculation.

The *Sclerotinia sclerotiorum* strain UF-1 was cultured on potato dextrose agar (PDA) medium for 3 days at 23 °C. Plugs of 5 mm in diameter were taken from the outer edge of the mycelium and placed, mycelial side down, on the main stem. The plugs were secured with breathable 3M medical tape and cling film to maintain moisture. Lesion length on the main stem was measured at 7 days post inoculation (dpi) using a measuring scale. The circumference of the main stem at the site of lesion formation was also recorded at 7 dpi.

### 4.2. Data Quality Control and Single-SNP GWAS Analysis

The SNP database of 300 oilseed rape germplasm accessions was obtained from the BnaSNPDB website database (https://bnapus-zju.com/bnasnpdb/, accessed on 1 June 2022) [76]. SNP calling was performed by mapping clean reads of each accession to “Darmor-bzh” reference genomes (B. napus v4.1 genome, http://www.genoscope.cns.fr/brassicanapus/data/, accessed on 5 June 2022). Quality control of SNPs was conducted using PLINK v1.9 [77], considering that SNPs were filtered based on genotype minor allele frequency (0.15), Hardy–Weinberg equilibrium (HWE) with a threshold of 1 × 10^−5^, and minor allele frequency (MAF) of 0.03. 2574368 high-quality SNPs were obtained based on the “Darmor-bzh” reference genome. LD decay calculation and plotting were performed using the PopLDdecay software v3.42 [78]. The LD heatmap was generated using the BnaSNPDB website. Broad-sense heritability (h^2^) was calculated using the inti package in R v4.3.0. Principal component analysis (PCA) analysis was conducted using the EIGENSOFT package v6.0.1 [79].

Single-SNP Genome-Wide Association Study (GWAS) was performed using the rMVP package v1.0.0 in R v4.3.0 [39]. Kinship was calculated using rMVP, and three models (GLM, MLM, FarmCPU) were compared. Manhattan plots and Q-Q plots were generated using rMVP to assess SNP associations and significance. Gene phenotype distribution plots and normality analyses were also conducted using rMVP. To conduct gene matching analysis, a nearby genomic region of around 20Kb surrounding the SNPs was selected.

### 4.3. RAL Identification and Enrichment Analysis

We identified significant SNPs from the single-SNP GWAS results by filtering (−log_10_(*p*_value) > 5). RALs associated with resistance to Ss were considered when there were at least 3 significant SNPs within 18 Mb. The distribution of these RALs on the chromosomes was visualized using MG2C [80]. *B. napus* genes were searched against the Gene Ontology terms (https://geneontology.org/, accessed on 15 September 2022), IPR database (http://pir.georgetown.edu/iproclass/, accessed on 15 September 2022), and ProSitePatterns database (http://www.ebi.ac.uk/interpro/, accessed on 15 September 2022). Enrichment analysis was performed using the OmicStudio tools (https://www.omicstudio.cn/, accessed on 20 September 2022) [81].

### 4.4. Hap-GWAS, WGCNA and DEGs Analysis

Hap-GWAS analysis was conducted using the R package RAINBOWR v0.1.36 [82], employing the parameters “window.size.half = 5” and “window.slide = 11”. For the WGCNA, RNA-seq data from the previous study [10] were utilized. The RNA-seq data were deposited in the NCBI Sequence Read Archive under the accession number SRP053361. WGCNA was performed using the OECloud tools (https://cloud.oebiotech.com, accessed on 6 September 2023). Differentially expressed genes between inoculated and mock-inoculated samples were identified based on strict criteria: an absolute value of log2 fold changes ≥ 1 and a false discovery rate (FDR) ≤ 0.01.

### 4.5. Machine Learning

All data related to resistance to Ss were collected and combined (RNA-seq and gene function annotations) with our dataset (WGCNA, single-SNP GWAS, and HAP-GWAS) to construct a training feature set. Protein sequences from the gene models were BLASTed against the TAIR 10 protein database to determine the gene annotation. The dataset consisted of 2001 positive samples and 1439 negative samples. We allocated 80% of the dataset for model training and the remaining 20% for model testing. The training sample had a feature dimension of 13,666, with 2 dimensions for WGCNA features, 7 dimensions for GWAS features, 2 dimensions for HAP GWAS, and 13,655 dimensions for gene function (GO and other database annotations).

For model selection, we compared four machine learning methods: LR, SVM, XGBoost, and RF. We utilized scikit-learn, a popular open-source machine learning library for Python, for data preprocessing, PCA, and model training. PCA was applied to retain 99.5% of the variance in the features, effectively reducing the feature dimension from 13,655 to 20 while preserving most of the feature variance. During model training, we performed a grid search to find the best parameters for all models, such as the number of estimators (n_estimator) in the range of (30, 40, 50, 60) and the maximum number of features. To evaluate the performance of the trained models, we utilized appropriate evaluation metrics such as accuracy, precision, recall, and F1 score. Additionally, techniques like cross-validation were employed to assess the generalization ability of the models.

### 4.6. Selective Sweep Scans

The vcftools software v4.1 [83] was used to perform selective sweep scans. Nucleotide diversity (π) was calculated with the parameters “--window-pi 20000 --window-pi-step 1000” to assess genetic variation within the population. Tajima’s *D* value (--TajimaD 10000) and Fst value (--fst-window-size 20,000 --fst-window-step 1000) were also computed to evaluate the occurrence of selection and population differentiation, respectively.

### 4.7. Statistical Analysis

Statistical analyses were conducted using GraphPad Prism 8 software. One-way ANOVA followed by Duncan’s new multiple range test (DMRT) was utilized for group comparisons. All data were presented as the mean ± standard deviation (SD).

## Figures and Tables

**Table 1:** Physical position of RALs associated with resistance to Ss.

RAL Name ^1^	Chr ^2^	Start	End	Recurring QTLs in the Reference	Reference
RSSA01a	chrA01	29,310	7,115,091		
RSSA01b	chrA01	13,304,776	22,353,741	chrA01:12,444,829–19,857,639	Wu et al., 2013 [9]
RSSA02a	chrA02	6,327,197	8,672,595	chrA02:5,418,060–7,389,046	Qasim et al., 2020 [40]
				chrA02:8,152,495–9,281,526	Qasim et al., 2020 [40]
RSSA02b	chrA02	17,499,716	19,951,552	chrA02:16,670,964–20,474,897	Wu et al., 2013 [9]
RSSA03a	chrA03	178,592	4,297,931	chrA03:2,702,453–4,262,421	Wu et al., 2019 [41]
RSSA03b	chrA03	8,896,717	11,278,534		
RSSA03c	chrA03	15,124,067	22,211,498	chrA03:15,547,362–16,064,878	Zhao et al., 2006 [42]
RSSA04a	chrA04	9,608,450	12,922,254		
RSSA04b	chrA04	14,872,047	18,447,087		
RSSA05	chrA05	17,966,325	20,397,420		
RSSA06a	chrA06	1,853,642	2,407,529		
RSSA06b	chrA06	6,152,638	8,944,061		
RSSA06c	chrA06	17,880,852	21,689,696	chrA06:20,965,425–23,324,273 chrA06:21,629,047–23,499,018	Wu et al., 2013 [9] Wu et al., 2019 [41]
RSSA07a	chrA07	10,082,564	14,250,889		
RSSA07b	chrA07	20,186,060	21,609,303		
RSSA09a	chrA09	6,448,538	6,596,656		
RSSA09b	chrA09	32,071,202	32,105,505	chrA09:28,638,676–31,720,464	Qasim et al., 2020 [40]
RSSA10a	chrA10	1,236,286	4,041,051		
RSSA10b	chrA10	10,486,133	14,839,131		
RSSAnn_random_a	chrAnn_random	15,679,614	21,810,062		
RSSAnn_random_b	chrAnn_random	25,598,042	33,228,887		
RSSAnn_random_c	chrAnn_random	39,652,449	46,732,598		
RSSC01	chrC01	19,283,445	34,365,756		
RSSC02a	chrC02	14,388,409	18,630,147		
RSSC02b	chrC02	26,296,409	28,749,960		
RSSC03a	chrC03	4,693,455	9,737,761		
RSSC03b	chrC03	20,346,765	33,132,785	chrC03:22,217,518–30,597,169 chrC03:30,597,169–47,861,855	Qasim et al., 2020 [40] Qasim et al., 2020 [40]
RSSC03_random	chrC03_random	1,845,659	1,847,988		
RSSC04a	chrC04	19,676,382	20,444,349	chrC04:11,691,778–28,720,453	Zhao et al., 2006 [42]
RSSC04b	chrC04	23,959,219	26,737,257	chrC04:11,691,778–28,720,453	Zhao et al., 2006 [42]
RSSC04c	chrC04	39,374,823	43,990,961	chrC04:40,419,964–41,916,428	Wu et al., 2016 [43]
RSSC05a	chrC05	1,079,675	8,269,010		
RSSC05b	chrC05	10,013,537	17,710,073		
RSSC05c	chrC05	33,795,591	39,750,069		
RSSC06a	chrC06	2,873,709	4,819,806		
RSSC06b	chrC06	12,871,211	17,346,441		
RSSC07a	chrC07	22,967,050	27,205,509		
RSSC07b	chrC07	30,140,825	33,700,961	chrC07:29,634,609–31,761,057	Wu et al., 2013 [9]
RSSC07c	chrC07	40,473,495	44,450,958		
RSSC08a	chrC08	14,471,405	23,488,895	chrC08:20,654,840–20,788,744	Wu et al., 2016 [43]
RSSC08b	chrC08	29,321,327	33,960,528	chrC08:31,404,544–32,040,944 chrC08:31,404,544–33,506,513	Wu et al., 2013 [9] Wu et al., 2013 [9]
RSSC09a	chrC09	3,611,793	21,444,314		
RSSC09b	chrC09	33,468,285	33,556,099		
RSSC09c	chrC09	41,569,762	45,686,680	chrC09:43,326,113–43,593,795	Zhao et al., 2006 [42]
RSSCnn_random_c	chrCnn_random	19,417,331	25,686,759		
RSSCnn_random_d	chrCnn_random	41,618,220	49,282,425		
RSSCnn_random_e	chrCnn_random	55,253,392	60,160,894		
RSSCnn_random_f	chrCnn_random	62,592,394	69,187,154		

^1^ RAL name: name of Resistance-Associated Loci. ^2^ Chr: Chromosome.

**Table 2:** Prediction of calcium signaling genes associated with Ss resistance on Chromosome A06 using iMAP.

RAL_Name ^1^	Gene_ID	Chr ^2^	Start	End	At_Gene ^3^	Symbol	Description	Score
RSSA06a	BnaA06g03950D	chrA06	2,392,927	2,389,029	AT1G48260	CIPK17	CBL interacting protein kinase 17	0.78
RSSA06b	BnaA06g12600D	chrA06	6,535,463	6,537,122	AT1G18480	SLP2	Calcineurin like metallo phosphoesterase superfamily protein	0.97
RSSA06b	BnaA06g12660D	chrA06	6,551,737	6,551,264	AT1G18530	CML15	Calmodulin-like 15	0.92
RSSA06b	BnaA06g13020D	chrA06	6,809,184	6,812,279	AT1G18840	IQD30	IQ domain 30	0.98
RSSA06b	BnaA06g14070D	chrA06	7,550,927	7,554,322	AT1G19870	IQD32	IQ domain 32	0.97
RSSA06b	BnaA06g15280D	chrA06	8,382,965	8,383,757	AT1G21550	CML44	Calcium binding EF hand family protein	1.00
RSSA06b	BnaA06g15970D	chrA06	8,783,588	8,786,720	AT5G24430	CPK4	Calcium dependent protein kinase	0.76

^1^ RAL_name: name of Resistance-Associated Loci. ^2^ Chr: Chromosome. ^3^ At_gene: Arabidopsis gene homologs of oilseed rape genes.

## Data Availability

The original contributions presented in this study are included in the article/supplementary material; further inquiries can be directed to the corresponding author.

## Supplementary Materials

The supporting information can be downloaded at: https://www.mdpi.com/article/10.3390/ijms25136932/s1.
