# Supplementary material for: Integrated Assays of Genome-Wide Association Study, Multi-Omics Co-Localization, and Machine Learning Associated Calcium Signaling Genes with Oilseed Rape Resistance to Sclerotinia sclerotiorum
Source: Int J Mol Sci. 2024 Jun 25;25(13):6932. doi: 10.3390/ijms25136932 (PMC11240920; doi:10.3390/ijms25136932)
Supplement: Supplementary file 1 [file ijms-25-06932-s001.zip › Supplimentary files-R1/Wang XY-GWAS_IJMS_Supplemental Figures_submitted.pdf]

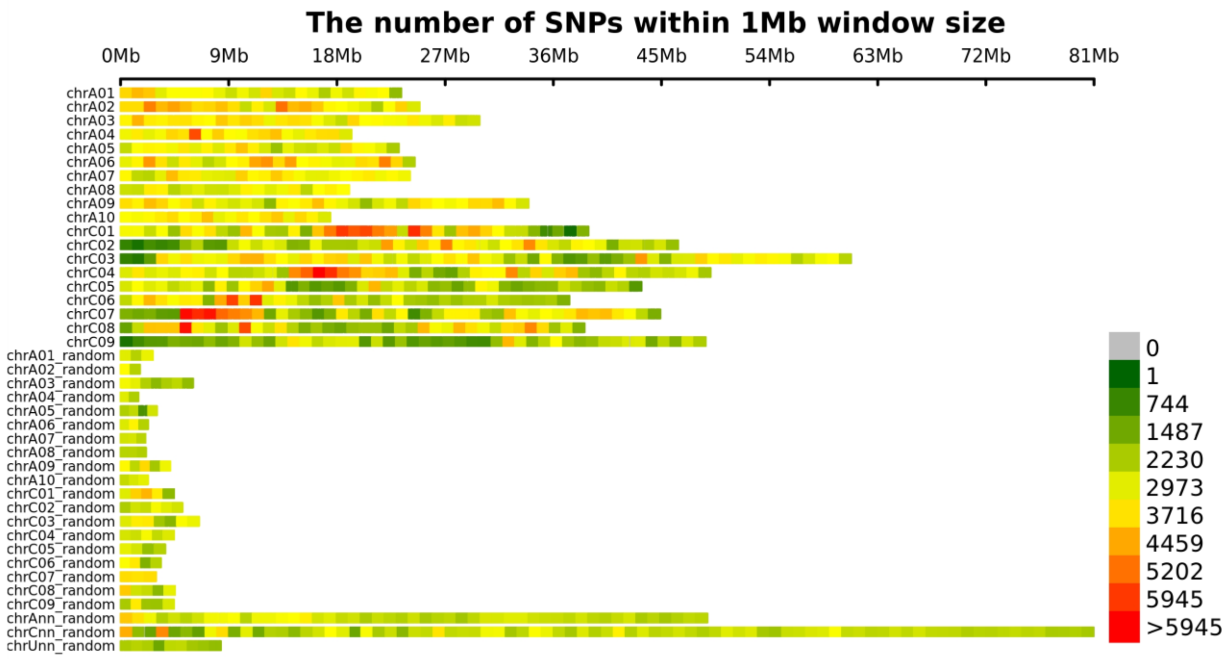

Figure S1 Single nucleotide polymorphism (SNP) density and distribution across the oilseed rape chromosomes.

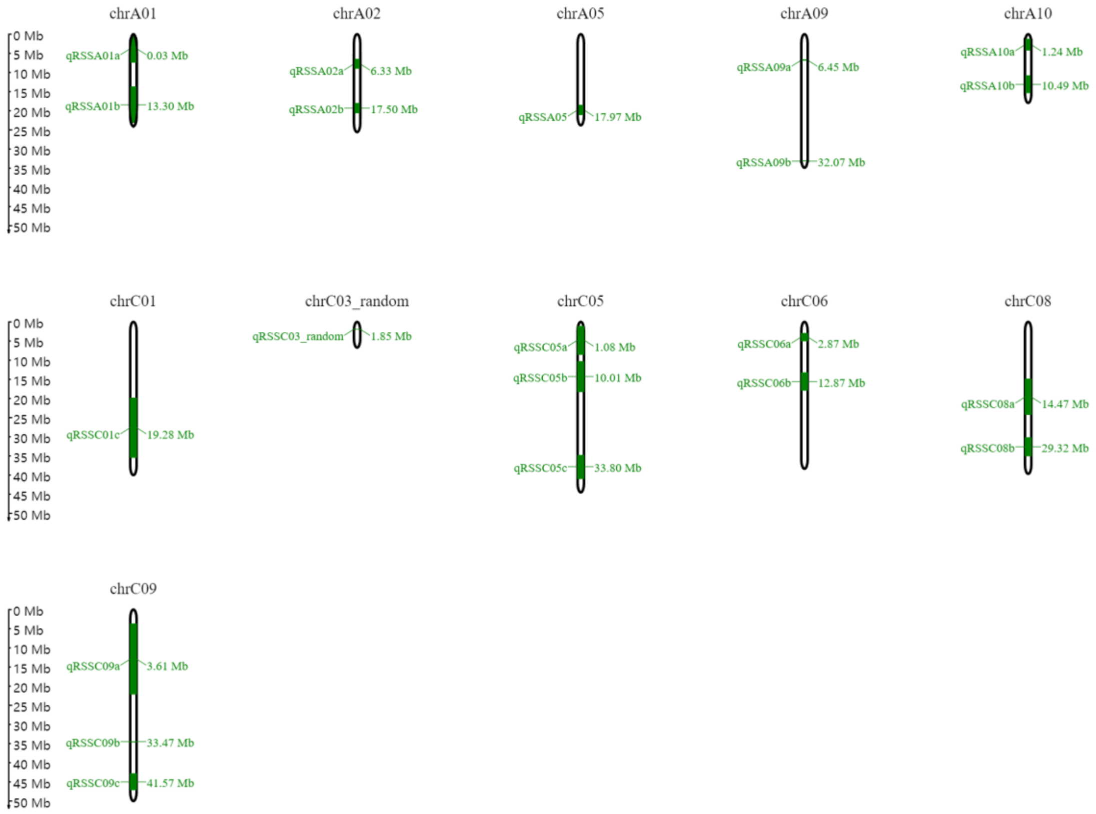

Figure S2. Chromosomal distribution of 22 out of 48 SSR resistance-associated RALs.

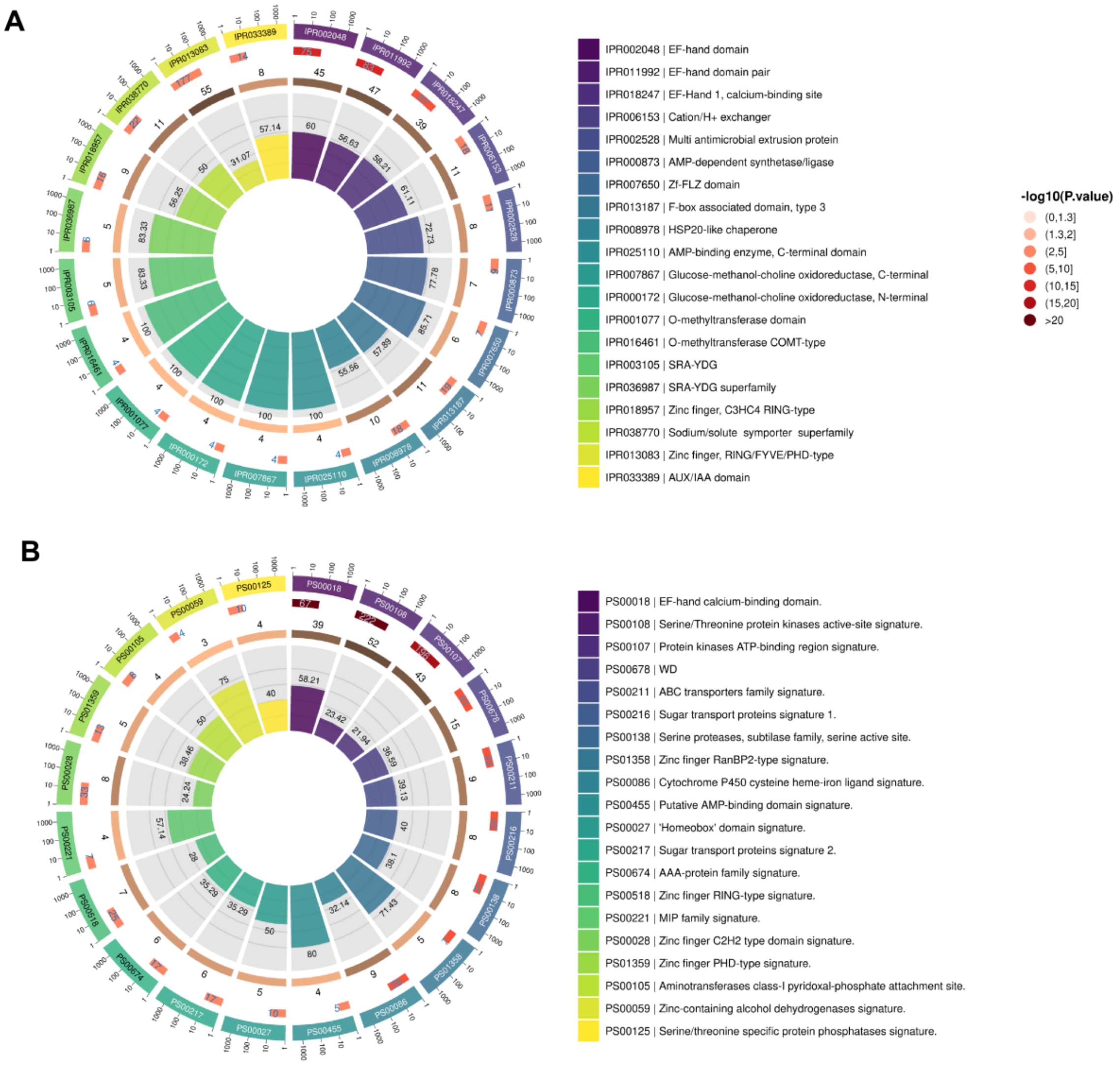

Figure S3. Gene function and enrichment analysis of LoopCircos results.

(A-B) Gene function and enrichment analysis of LoopCircos results in IPR database (A) and ProSitePatterns database (B).

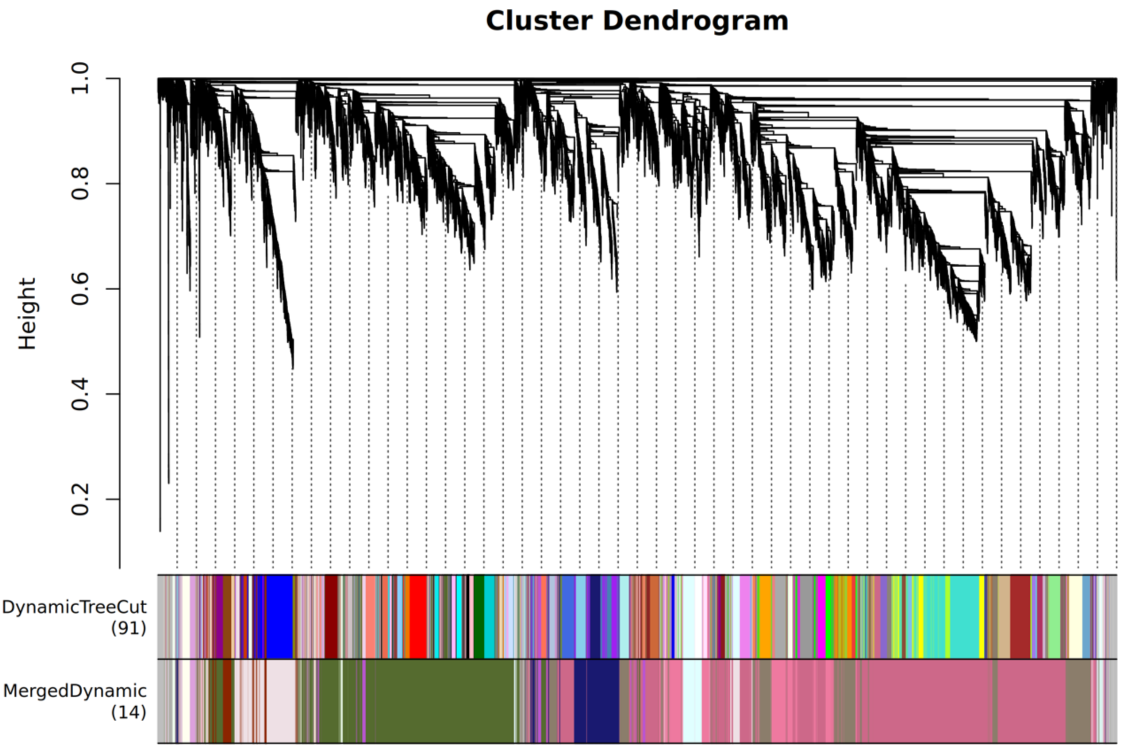

Figure S4. Clustering tree of the WGCNA analysis.

The gene clustering tree constructed using the dissTOM matrix generated by WGANC method, which incorporates weighted correlations. The lower section of the figure shows the distribution of genes within each module.

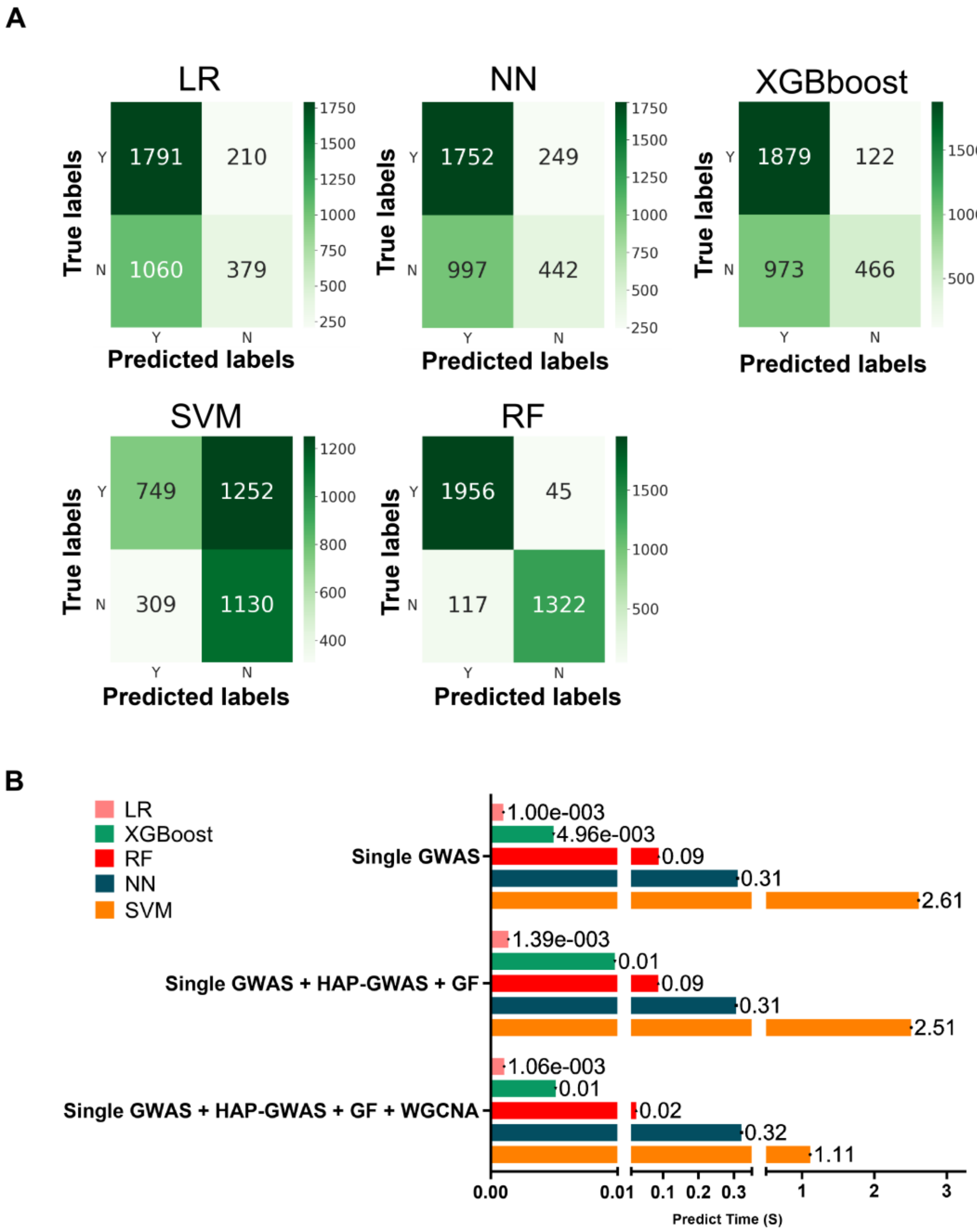

Figure S5. Assessment of algorithms and feature sets for genomic analysis in predictive modeling. (A) Confusion Matrix of LR, NN, XGBoost,, SVM and RF algorithms using Single GWAS + HAP-GWAS + GF as the feature set.

(B) Performance comparison of LR, XGBoost, RF, NN and SVM algorithms in terms of Predict Time using different feature sets, including Single GWAS, Single GWAS + HAP-GWAS + GF, and Single GWAS + HAP-GWAS + GF + WGCNA for machine learning.
